# Parental correlates in child and adolescent physical activity: a meta-analysis

**DOI:** 10.1186/s12966-015-0163-y

**Published:** 2015-02-11

**Authors:** Christopher A Yao, Ryan E Rhodes

**Affiliations:** Exercise Science, Physical and Health Education, University of Victoria, PO Box 3010 STN CSC, Victoria, V8W 3N4 Canada

**Keywords:** Preschool, Childhood, Adolescence, Physical activity, Parental support, Parental role modeling, Parental behaviours, Meta-analysis, Review

## Abstract

**Objective:**

Physical activity (PA) has a profound impact on health and development in children. Parental behaviors (i.e., modeling and support) represent an obvious important factor in child PA. The purpose of this paper was to provide a comprehensive meta-analysis that overcomes the limitations of prior narrative reviews and quantitative reviews with small samples.

**Methods:**

Ten major databases were used in the literature search. One-hundred and fifteen studies passed the eligibility criteria. Both fixed and random effects models with correction for sampling and measurement error were examined in the analysis. Moderator analyses investigating the effects of child’s developmental age, study design, parental gender, measurement of child PA, and quality rating were performed.

**Results:**

Based on the random effects model, the results showed that parental modeling was weakly associated with child PA (summary *r* = .16, 95% CI .09-.24) and none of the proposed moderators were significant. Separate analyses examining the moderating effects of parental gender and boys’ PA found that that father-son PA modeling (*r* = .29, 95% CI .21-.36) was significantly higher compared to mother-son PA (*r* = .19, 95% CI .14-.23; p < .05). However, parental gender did not moderate the relationship between parental modeling and girls’ PA (*p* > .05). The random effects model indicated an overall moderate effect size for the parental support and child PA relationship (summary *r* = .38, 95% CI .30-.46). Here, the only significant moderating variable was the measurement of child PA (objective: *r* = .20, 95% CI .13-.26; reported: *r* = .46, 95% CI .37-.55; *p* < .01).

**Conclusions:**

Parental support and modeling relate to child PA, yet our results revealed a significant degree of heterogeneity among the studies that could not be explained well by our proposed moderators.

It has been widely acknowledged by health researchers that participation in regular physical activity (PA) is linked to various health benefits and prevention of chronic disease. In spite of the overwhelming evidence that supports an association between PA and health, much of the populace does not commensurate with the national recommendations. Particularly, many children in North America are insufficiently active to reap the health benefits associated with regular PA. A recent Canadian national survey estimated that 9% of boys and 4% of girls between the ages of six to nineteen met the current recommendations [1]. Likewise, data from the United States showed that more than half of the children surveyed were insufficiently active [2]. At this juncture, intervention efforts to improve child PA levels have produced very modest results [3]. Thus moving forward, it will be crucial to properly identify the key correlates in child and adolescent PA to further the planning and development of PA interventions [4].

Presently, a total of 14 review papers [5-18] and three reviews of reviews [19-21] have been published in this area. From these reviews, parental modeling of PA and parental support of child PA have emerged as major themes. However, many of these reviews have discordant findings. For instance, 12 review papers examining the relationship between parent and child PA have shown variable results [5-9,12-14,16,19-21]. Three of the 12 reviews do not support a link between parent PA and child PA [14,20,21], while eight reviews have suggested the association as inconclusive [5-7,9,12,13,17,19]. Unlike the findings for parental modeling and child PA, parental support has emerged as a consistent correlate of child and adolescent PA in a number of narrative reviews [6-9,11,12,14,16,18-21]. The more striking absence in this theme is the limited quantitative synthesis in order to provide a point-estimate of the parental support-PA relationship. Only one meta-analysis has examined parental support (r = .23), but it is several years old and was restricted to three studies [8].

Another pertinent issue that surrounds parental support as a correlate of child PA has been how support has been defined and measured. Parental support has often been measured as an omnibus of various support behaviours and has no consistent set of behaviours [22]. In some cases, researchers have grouped and measured multiple support behaviours as tangible (e.g., providing transportation, financial support) and intangible forms of support (e.g., praise and encouragement). Through these forms of measurement, it is unclear to which specific individual support behaviours may be important in child PA. A more comprehensive synthesis of these support factors is needed.

Finally, prior reviews on this topic have been restricted to very specific age-ranges, which reduces our understanding to whether modeling and support vary across the developmental spectrum. No prior meta-analyses have explored the parental correlates according to developmental stages (i.e., preschool, childhood, and adolescence). A meta-analysis is necessary to consolidate and clarify the overall information.

With these limitations in mind, the aim of this meta-analysis was to provide a cohesive and comprehensive examination of the parental correlates, and potential moderators, of child PA. Here, the five postulated moderators included the child’s developmental age, method in which child PA is measured (objective or reported), geographical location of the sample population, study design, and quality of the study. Moreover, we investigated the possibility of intergenerational gender interactions between parent and child behaviours. It was hypothesized that overall parental PA would have a negligible to small correlation with child and adolescent PA, explaining the prior inconsistencies among the narrative reviews; whereas overall parental support will have a small to medium correlation. Among the individual support behaviours, it was postulated that a small effect size will be found for the various support behaviours and child PA. Our analysis of intergenerational gender interactions between parental and child was considered exploratory.

## Methods

### Eligibility criteria

To ensure transparency and complete reporting, the protocols for this study were in accordance to the recommendations put forth by the PRISMA statement for conducting systematic reviews and meta-analyses [23]. Studies were included if: 1) children were between 2.5 and 18.0 years; 2) an assessment of parental/family support, individual parental support behaviour(s), or parental PA as the independent variable; 3) a measurement of children’s PA as the dependent variable; and 4) an effect size illustrating the relationship between independent and dependent variables or the availability of statistics to calculate an effect size (e.g., means and standard deviation). Studies were excluded from the review if: 1) social support measures consolidated parental sources with teachers, peers, or friends; 2) the study was qualitative; and 3) not published in English.

PA was defined as “any bodily movement produced by skeletal muscles that results in energy expenditure” [24]. This definition encompassed both structured (e.g., organized sports, lessons) and unstructured PA (e.g.. leisure-time PA, play). Encouragement to be active, parent–child co-activity, praising the child’s activity, watching the child be active, informing the child that they are performing well, telling the child that PA is beneficial, and providing transportation to PA venues were classified as parental support behaviours. Other behaviours such as supplying the child with PA equipment and financial support, and enrolling the child in PA programs were classified as individual parent support behaviours.

### Search strategy

**Figure 1:**
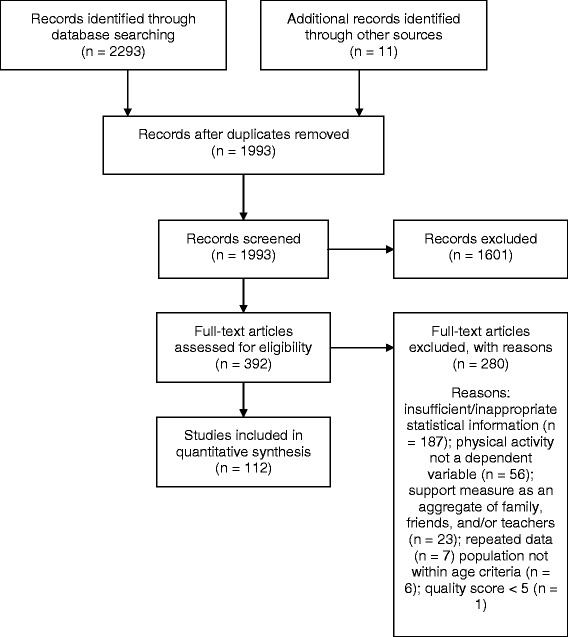
**PRISMA flow-chart.**

Publications from January 1970 to November 2014 were systematically reviewed for this paper (Figure 1). Ten databases were used to locate relevant articles: EBSCO (Academic Search Complete, Academic Search Premier, CINAHL, Health Source, MEDLINE, PsycINFO, Social Sciences, SPORTDiscus), PubMed, and ISI Web of Science. The following key terms were used: physical activity, exercise, sport, adolescent, youth, children, preschool, parental support, parental physical activity, role modeling, parental influence, and parental correlates. One author conducted the search and manually cross-referenced studies to ensure saturation of the literature. The eligibility criteria and search strategy followed a protocol used in previously published meta-analyses and reviews [25,26]. The reference sections of reviews and individual studies were carefully inspected to locate any additional publications.

### Screening

Using the inclusion criteria previously established by both reviewers, one reviewer initially screened citations based on the title and abstract. Potentially relevant abstracts were selected and the full article was located if it was deemed suitable for the study. A full consensus by the two reviewers was required in order for the studies to be included in the analysis.

### Data abstraction

Information regarding authors, publication year, country, sample (number of participants, age, gender), study design (cross-sectional/prospective), measurement tools (i.e., PA and social support measures), reliability of the measures, parental gender, and reported effect sizes, were abstracted onto a Word document. Once the coded data was entered, the file was imported into the Comprehensive Meta-Analysis version 2 program for further analyses [27].

### Analyses

Based on the hypothesized moderators, the studies included in the analysis were categorized and coded by developmental age (preschool 2–5.4 yrs, childhood 5.5-12.4 yrs, adolescence 12.5-18 yrs), geographical location (Australia & New Zealand, Asia, Canada, Europe, USA), study design (cross-sectional, prospective), type of PA measure used to determine child PA (objective: accelerometer, pedometer, heart rate monitor; reported), and quality (high, moderate, low). Upon further investigation of previous meta-analyses and reviews, some of the studies included did not appropriately categorize effect sizes that represented the overall effect sizes for parental-child PA variables. For instance, samples only examining girls’ or boys’ PA were previously amalgamated into overall child associations rather than conducted in separate analyses. In our analyses, the correlates for boys, girls, and mixed samples were abstracted, categorized, and analyzed separately.

In the case that more than one type of PA measure was reported (ex. overall PA levels versus moderate to vigorous PA), the variable that best reflected the national recommendations for PA (i.e., moderate to vigorous PA) was incorporated into the analysis. Studies that incorporated a family support measure were included in the analysis.

To assess the potential risk of bias and methodological quality, each study was critically appraised using an adapted version of Downs and Black’s [28] 22-item assessment tool. This modified tool is comparable to the Cochrane Collaboration’s instrument for assessing risk of bias and has been used in several published reviews [25,26,29]. For the purposes of this study, items from the original checklist pertaining to experimental studies and items that were not applicable to this study were excluded. The adapted version utilized a 14-point scoring scheme, where each item was scored one point based on a yes (1) or no (0) response. Studies scoring 12–14 points were deemed high-quality studies, 8–11 points were regarded as moderate-quality studies, and lower quality studies were below 7 points. Studies that scored 4 points or less were excluded.

Effect sizes included in the analysis were further corrected for sample size and attenuated for potential measurement error. Correction of measurement error procedures was based on the reported reliabilities of the measures found in the study. In the case that the reliability of the measure was not detailed, an rxy = .70 was used. Based on previous publications, this reliability has been identified as a conservative, yet acceptable estimate for reliability [30]. For accelerometer measures that have obtained 4–9 days of data, the recommended reliability estimate of .80 was used [31]. No subsequent correction procedures were conducted for effect sizes derived from structural equation models or hierarchical linear models as these forms of analyses account for measurement error.

Both fixed and random effects models were used to determine the overall effect sizes for both uncorrected and corrected effect sizes. However, only corrected effect sizes from the random effects model will be discussed. The strength of the correlation was categorized based on Cohen’s recommendations [32]. According to these guidelines, a correlation of .09 or less was considered as a null effect, .10 a small effect, .30 a medium effect, and .50 a large effect. In addition to the overall effect sizes, 95% confidence intervals were calculated. To determine heterogeneity among the effect sizes, a Q-statistic and I^2^ was computed. The Q-statistic identifies whether the observed variance in effect sizes is no greater than that expected by sampling error alone, whereas the I^2^ denotes the dispersion. For the purposes of this study, I^2^ values of 25 were categorized as having a low dispersal, 50 as a moderate dispersal, and 75 as a high dispersal. Moderator analyses investigating the effects of child’s developmental age, study design, parental gender, measurement of child PA, and quality rating were performed using the corrected r’s with fixed and random effects models. A minimum of 4 studies was required in each moderator analysis to deem it as a valid moderator. To identify the correlations between the intergenerational relationships between parent and child, separate analyses were used to examine whether the parents’ gender moderated boys’ and girls’ PA. To assess the extent of publication bias in our samples, Rosenthal’s classic fail-safe N [33] and Duval and Tweedie’s Trim and Fill procedures [34,35] were conducted. All data was analyzed in February 2013 using Comprehensive Meta-Analysis.

## Results

A total of 2,293 potentially relevant citations were identified in the initial search. The screening procedures resulted in a total of 112 studies, with 11 studies extracted from the reference listing of the included articles (see Figure 1). Table 1 describes the characteristics of the 115 independent samples included for the investigation. Details of the included studies are presented in Tables 2,3,4,5,6 and 7. Duplicated studies were not included in the analysis.

**Table 1:** **Descriptive statistics of 112 studies investigating parental factors and child and adolescent physical activity (n = 115 independent samples)**

**Characteristic**	**Samples n (%)**
Geographical location	
Asia	4 (3)
Australia & New Zealand	11 (10)
Canada	8 (7)
Europe	31 (27)
South America	2 (2)
United States	59 (51)
Study design	
Cross-sectional	94 (82)
Prospective	21 (18)
Physical activity measurement	
Objective	31 (27)
Self-report	84 (73)
Quality rating	
High	18 (16)
Moderate	84 (73)
Low	13 (11)
Developmental age	
Preschool (2–5.4 yrs)	14 (12)
Childhood (5.5-12.4 yrs)	54 (47)
Adolescence (12.5-19.0 yrs)	47 (41)

**Table 2:** **Studies and effect sizes of parental modeling and child and adolescent physical activity (k = 36)**

**Study, country**	**Sample (number, gender, mean age)**	**Design**	**Parental PA measure**	**Child PA measure**	**Results**	**Corrected effect size**
Alderman et al. (2010) [36] USA	N = 70	PRO (1–9 yrs)	Parent self-report	Parent report	Children’s MVPA & parental PA: r = .44, p < .05 at baseline; r = .08, p < .05 at follow-up	.37
	43 m, 26 f		.70* (97% respondents mothers)	.70*		
	4-6 yrs at baseline; 5–15 yrs at follow-up				**Mean r = .26**	
Ammouri et al. (2007) [37] USA	N = 284	CS	Parent self-report	Child self-report	Adolescents’ PA & parental PA: β = .019	.03
	98 m, 186 f		GLTEQ	SAPAC		
	15.3 yrs		.70*	.80		
Berge et al. (2014) [38] USA	N = 200	CS	Parent self-report	Child self-report	Adolescents’ MVPA & parental MVPA β = .11, p < .05 (resident parent)	.15
	80 m, 120 f		GLTEQ	GLTEQ		
	14.2 yrs		.75 (80% resident parent mothers)	.72		
Dempsey et al. l(1993) [39] USA	N = 71	CS	Parent self-report	Child self-report	Children’s MVPA & parents’ MVPA: β = −.17	-.24
	36 m, 35 f		Adapted GLTEQ	Adapted GLTEQ		
	10.2 yrs		.70*	.70*		
Dowda et al. (2011) [40] USA	N = 369	CS	Parent self-report	Accelerometer (2 wks)	Children’s MVPA & parents’ PA: β = .002	.00
	179m, 194 f		Sport PA .67	.80*		
	4.2 yrs		Non-sport PA .71	Direct observation		
			(92% respondents mothers)	(OSRAC-P) Inter-observer .91		
Dzewaltowski et al. (2008) [41] USA	N = 57	CS	Child reported (adapted from the YRBSQ)	Child self-report	Children’s MVPA & parental PA: b = .22	.29
	18 m, 37 f			PDPAR		
	12.4 yrs		.90	ICC = .64		
Fredricks & Eccles (2005) [42] USA	N = 364	PRO (1 yr)	Parent self-report	Child self-report	Children’s sports PA & parents’ PA: r = .05 at baseline; r = .04 at follow-up	.06
	184 m, 180 f			Sports participation		
	Ages 7.0-11.0 yrs at baseline			.70*	**Mean r = .045**	
Heitzler et al. (2010) [43] USA	N = 720	CS	Parent self-report	Accelerometer (7 d)	Adolescents’ MVPA & parent PA: r = .07	.09
	352 m, 368 f		IPAQ	.80*		
	14.7 yrs		.70*			
Hendrie et al. (2011) [44] Australia	N = 106	CS	Parent self-report	Parent report	Children’s MVPA & parental PA: r = .145; partial r = .145	.19
	51 m, 55 f		Adapted from the Family Food Questionnaire α = .877 (92% respondents mothers)	CLASS		
	8.3 yrs			.70*		
Hennessy et al. (2010) [45] USA	N = 76	CS	Parent self-report	Accelerometer (5 d) .80*	Children’s MVPA & parental explicit modeling: β = −.04, p = .70	-.06
	26 m, 50 f		ICC = .55 (96% respondents mothers)			
	9.1 yrs					
Keresztes et al. (2008) [46] Hungary	N = 548	CS	Child report	Child self-report	Children’s MVPA & parents’ PA: OR = 2.10, 95% CI = 1.15-3.80	.41
	301 m, 247 f		.70*	.70*		
	12.2 yrs					
Labree et al. (2014) [47] Netherlands	N = 1943	CS	Parent self-report	Parent report	Children’s PA & parental modeling: r = .12, p < .05	.17
	970 m, 973 f		SQUASH	.70*		
	8.4 yrs		.70* (mostly mothers)			
Lei et al. (2004) [48] Taiwan	N = 798	CS	Child report	Child report	Adolescent MVPA & parental modeling: r = −.018, p = .616	-.02
	Age range: 12–18 yrs		Parent Socialization Scale	7-day PA Survey		
			.70*	.82		
Loprinzi et al. (2010) [49] Austrailia	N = 156	CS	Parent self-report	Parent report	Child PA & parents’ PA: β = −.04, p = .64	-.06
	75 m, 81 f		IPAQ	PAEC-Q		
	3.7 yrs		.70*	.70*		
Loprinzi et al. (2013) [50] USA	N = 176	CS	Parent self-report	Parent report	Children’s MVPA & parent PA: β = .17, p < .05	.24
	82 m, 94 f		IPAQ	PAEC-Q		
	4.0 yrs		.70* (85% respondents mothers)	.70*		
McMurray et al. (1993) [51] USA	N = 1253	CS	Parent self-report	Child self-report	Children’s PA & parents’ exercise habits: r = .006, p = .845	.01
	589 m, 664 f		.70* (70% respondents mothers)	.70*		
	8.8 yrs					
Moore et al. (1991) [52] USA	N = 100	CS	Accelerometer (5 d)	Accelerometer (5 d)	Children’s PA & parents’ PA: OR 3.5, 95% CI 1.2-9.8; r = .46	.66
	63 m, 37 f		.70*	.70*		
	10.4 yrs					
Mota (1998) [53] Portugal	N = 45	CS	Parent self-report	Child self-report	Children’s PA & parents’ VPA: r = .14	.17
			.88	.72	Children’s PA & parents’ MPA: r = .13	
	18 m, 27 f					
	10.1 yrs				**Mean r = .135**	
Østbye et al. (2013) [54] USA	N = 208	CS	Parent report	Accelerometer (7 d)	Children’s PA & parental modeling: r = .12	.15
	116 m, 92 f		Role modeling of PA	.80*		
			α = .80 (all mothers)			
	2-5 yrs					
Patnode et al. (2010) [55] USA	N = 294	CS	Parent self-report	Accelerometer (7 d)	Adolescents’ MVPA & parents’ PA: r = .003	.00
	149 m, 145 f		IPAQ	.80*		
	15.4 yrs		.70*			
Perusse et al. (1989) [56] Canada	N = 1610	CS	Parent self-report	Child self-report	Adolescents’ PA (exercise) & parental PA: r = .09, p < .05 (n = 1039)	.10
	14.6 yrs		3-day activity record	3-day activity record		
			.97	.91		
Pfeiffer et al. (2009) [57] USA	N = 331	CS	Parent self-report	Accelerometer (8–10 d)	Children’s MVPA & parents’ PA: r = −.04	-.05
	169 m, 162 f		.78 (94% respondents mothers)	.80*		
	4.3 yrs					
Poest et al. (1989) [58] USA	N = 514	CS	Parent self-report	Teacher report	Children’s PA & parents’ PA: r = .28, p = .045	.40
	269 m, 245 f		.70*	.70*		
	Preschool children					
Polley et al. (2005) [59] USA	N = 87	CS	Parent self-report	Child self-report	Children’s PA & parents’ PA: r = .11	.16
	Children		.70*	.70*		
Ruiz et al. (2011) [60] USA	N = 106	CS	Accelerometer (7 d)	Accelerometer (7 d)	Children’s MPA & parents’ PA: r = .739, p < .0001	.59
	52 m, 54 f		.70* (97.2% respondents mothers)	.80*	Children’s VPA & parents’ PA: r = − .07, p > .05	
	4.2 yrs				**Mean r = .4128**	
Rutkowski et al. (2012) [61] USA	N = 94	CS	Parent self-report	Child self-report	Adolescents’ MPVA & parental PA: r = −.23, p < .05	.29
	56 m, 28 f		IPAQ	PACE + MVPA		
	12.8 yrs		α = .80	ICC = .81		
Sallis et al. (1988) [62] USA	N = 33	PRO (2.5 yrs)	Parent self-report	FATS	Children’s MPA & parents’ PA: β = .53, p < .01	.70
	13 m, 20 f		.70*	.81		
	3.9 yrs					
Singh et al. (2009) [63] USA	N = 68288	CS	Parent report	Parent report	Children’s VPA & parents’ PA: r = .24*	.34
	Age range: 6–17 yrs		.70*	.70*	*controlled for other covariates	
Trost et al. (2003) [64] USA	N = 380	CS	Parent self-report	Child self-report	Adolescents’ PA & parental PA: β = .05, p = .28	.06
	171 m, 209 f		Test-retest	.79		
	14.0 yrs		.78			
Vella et al. (2014) [65] Australia	N = 4042	PRO (2 yrs)	Parent self-report MVPA	Parent report	Children’s PA & parental MVPA: OR = 1.03, 95% CI 1.01-1.05, p < .05; r = .01	.01
	2069 m, 1973 f		.70* (96% respondents mothers)	Organized sports participation		
	8.3 yrs					
				.70*		
Welk et al. (2003) [66] USA	N = 994	CS	Parent self-report	Child self-report	Children’s PA & parent PA: r = .28	.38
	505 m, 489 f		.68 children	PAQ-C		
	10.0 yrs		.68 boys	.75-.82		
			.67 girls			
			(82% respondents mothers)			
Williams & Mummery (2011) [67] Australia	N = 295	CS	Parent report	Child self-report	Adolescents’ MVPA & parents’ MVPA: adjusted OR = 0.59, 95% CI 0.29-1.20	-.29
	111 m 184 f		Active Australia Survey	APARQ		
	15.1 yrs		.70* (67% respondents mothers)	.70*		
Zecevic et al. (2010) [68] Canada	N = 102	CS	Parent self-report	Parental report	Children’s PA & parental PA habits: OR = 1.620, p < .10; r = .1874	.27
	54 m, 48 f		.70* (96% respondents mothers)	.70*		
	3.8 yrs					
Zhao & Settles (2014) [69] USA	N = 1514	CS	Parent self-report	Parent report	Children’s MPA & parental PA: β = −.15, p < .05	-.17
	763 m, 751 f		.70*	.70*	Children’s VPA & parental PA: β = −.09	
	11.8 yrs					
					**Mean β = −.12**	
Ziviani et al. (2005) [70] Australia	N = 50	CS	Parent self-report	Pedometer (4 d)	Children’s’ PA & parents’ PA: β = .23	.28
	26 m, 24 f		.97-1.00	.70*		
	7.7 yrs					
Ziviani et al. (2008) [71] Australia	N = 59	CS	Parent self-report	Pedometer (4 d)	Children’s PA (weekday) & parents’ PA: r = .06	.16
	26 m, 33 f		.97-1.00	.70*	Children’s PA (weekend) & parents’ PA: r = .21	
	8.9 yrs					
					**Mean r = .135**	

*Note.* *reliability not reported; APARQ = Adolescent Physical Activity Recall Questionnaire; CLASS = Children’s Leisure Activities Study Survey; CS = cross-sectional; d = days; f = female; FATS = Fargo Activity Timesampling Survey; GLTEQ = Godin Leisure-Time Exercise Questionnaire; IPAQ = International Physical Activity Questionnaire; PRO = prospective; m = male; MPA = moderate physical activity; MVPA = moderate-to-vigorous physical activity; OSRAC-P = Observational System for Recording Physical Activity in Children-Preschool Version; PA = physical activity; PAEC-Q = Physical Activity and Exercise Questionnaire for Children; SAPAC = Self-Administered Physical Activity Checklist; SQUASH = Short Questionnaire to Assess Health-Enhancing Physical activity; VPA = vigorous physical activity.

**Table 3:** **Studies and effect sizes for parental support and child and adolescent physical activity (k = 34)**

**Study, country**	**Sample (number, gender, mean age)**	**Design**	**Parental support measure**	**Child physical activity measure**	**Results**	**Corrected effect size**
Barr-Anderson et al. (2010) [72] USA	N = 73	CS	Child report	Child self-report	Children’s MVPA and perceived parental support: β = .17, p < .05	.24
	18 m, 55 f		Parental Support – aggregated measure (encouragement, coactivity, transportation, watching, inform)	Adapted GLTEQ		
	10.1 yrs		Child test-retest .88	Hard/strenuous test-retest .63		
				Moderate test-retest .52		
Davison et al. (2012) [73] USA	N = 767	CS	Parent report	Child self-report	Children’s MVPA &parental support: r = .20, p < .01 (n = 355)	.27
	392 m, 375 f		Parental support – aggregated measure (logistic support, modeling, co-activity, encouragement)	.70*	Adolescent’s MVPA & parental support: r = .36, p < .01 (n = 412)	.49
	Age range: 6.0-12.0 yrs & 13.0-19.0 yrs		α = .78			
Dowda et al. (2011) [40] USA	N = 369	CS	Parent report	Accelerometer (2 wk)	Children’s MVPA & parental support: β = .28	.34
			Parental support – aggregated measure (encourage, coactivity, transportation, watching child, providing information)	.80*		
	175 m, 194 f			Direct observation (OSRAC-P)		
	4.2 yrs					
			Test-retest .81 (92% respondents mothers)	Inter-observer .91		
Hagger et al. (2009) [74]	N = 840	PRO (5 wks)	Child report	Child self-report	Children’s MVPA (UK; n = 210) & parental support: r = .47, p < .01	.55
4 countries: UK, Estonia, Finland, Hungary	380 m, 460 f		Parental support – aggregated measure (provision of opportunities, choices, and options to be active)	Adapted GLTEQ	Children’s MVPA (Estonia; n = 268) & parental support: r = .36, p < .01	.45
	Age range: 13.2-15.0 yrs		UK α = .96	UK .77	Children’s MVPA (Finland; n = 127) & parental support: r = .41, p < .01	.51
			Estonian α = .94	Estonian .68	Children’s MVPA (Hungary; n = 235) & parental support: r = .20, p < .01	.26
			Finland α = .96	Finland .67		
			Hungary α = .90	Hungary .67		
Hamilton & White (2008) [75] Australia	N = 423	CS	Child report	Child self-report	Adolescents’ MVPA & parental support: r = .37, p < .001	.53
	172 m, 251 f		Parental support – aggregated measure (co-activity, watch, encouragement, praise, transportation)	.70*		
	13.5 yrs		.70*			
Heitzler et al. (2006) [76] USA	N = 3114	CS	Parent reported	Child self-report	Children’s organized PA & parental support: OR = 1.65, 95% CI 1.45-1.88, p < .001	.30
	Age range: 9.0-13.0 yrs		Parental support – aggregated and individually reported (coactivity, watching child, & transportation)	Test-retest .64		
			Test-retest .65			
Heitzler et al. (2010) [43] USA	N = 720	CS	Child report	Accelerometer (7 d)	Adolescents’ MVPA & parental support: r = .19, p < .05	.24
	352 m, 268 f		Parental support – aggregated measure (encouragement, coactivity, watch, praise) α = .80	.80*		
	14.7 yrs					
Hendrie et al. (2011) [44] Australia	N = 106	CS	Parent report	Child self-report	Children’s MVPA & parental support: r = .162; r = .18 when controlled for parent demographic factors)	.22
	51 m, 55 f		Parental support – aggregate measure (watching, transportation)	CLASS		
	8.3 yrs			.70*		
			α = .79 (92% respondents mothers)			
Hennessy et al. (2010) [45] USA	N = 76	CS	Parent report	Accelerometer (5 d)	Children’s PA & logistical support: β = .18, p = .12	.26
	26 m, 50 f		Logistical support	.70*		
	9.1 yrs		α = .67			
Kim & Cardinal (2010) [77] Korea	N = 1347	CS	Child report	Child self-report	Adolescent PA & parental support: r = .19, p < .01	.22
	943 m, 404 f		Parental support – aggregated measure (e.g., encouragement)	GLTEQ		
	16.4 yrs			Test-retest .86		
			test-retest .83			
Labree et al. (2014) [47] Netherlands	N = 1943	CS	Parent report	Parent report	Children’s PA & parental support: r = .21, p < .05	.31
	970 m, 973 f		Parental support	.70*		
	8.4 yrs		α = .64 (respondents predominantly mothers)			
Langer et al. (2014) [78] USA	N = 421	CS	Parent report	Accelerometer (7 d)	Children’s MVPA & parental support: r = .20, p < .001	.25
	213 m, 208 f		Parental support – aggregated measure (encouragement, co-activity, transportation, watch)	.80*		
	6.9 yrs		α = .77 (93% respondents mothers)			
Lawman & Wilson (2014) [79] USA	N = 181	CS	Child report	Accelerometer (7 d)	Adolescent’s MVPA & parental support: r = .09	.11
	72 m, 109 f		Parental support – aggregated measure	.80*		
	13.3 yrs		α = .89			
Lei et al. (2004) [48] Taiwan	N = 798	CS	Child report	Child report	Children’s MVPA & parental support: r = .12, p < .001	.17
	Age range: 12–18 yrs		Parental support	7-day PA survey		
			.75	.70*		
Loprinzi & Trost (2010) [49] Australia	N = 156	CS	Parent report	Parent report	Children’s PA (at home) & parental support: β = .16, p < .05	.12
	75 m, 81 f		Parental support – aggregated measure (encourage, co-activity, transportation, watch, inform)	PAEC-Q (PA at home)	Children’s PA (daycare) & parental support: β = .01	
	3.7 yrs		test-retest .81	.70*	**Mean β = .09**	
				Accelerometer (2 d)		
				(PA during daycare)		
				.70*		
Loprinzi et al. (2013) [49] USA	N = 176	CS	Parent report	Parent report	Children’s MVPA & parental support: β = .29, p < .05	.40
			Parental support – aggregated measure (encourage, co-activity, transportation, watch, inform)	PAEC-Q		
	82 m, 94 f					
	4.0 yrs					
			α = .75 (85% respondents mothers)	.70*		
Ommundsen et al. (2006) [80] Norway	N = 760	CS	Child report	Child self-report	Children’s PA & parental support: r = .40, p < .001	.57
	379 m, 381 f		Parental support - aggregated measure (encouragement, co-activity)	PEACH		
	9.0 & 15 yr olds		.70*	.70*		
Østbyte et al. (2013) [54] USA	N = 208	CS	Parent report	Accelerometer (7 d)	Children’s PA & parental support: r = .26, p < .05	.34
	116 m, 92 f		Parental support – aggregated measure	.80*		
	2-5 yrs		α = .75 (all mothers)			
Patnode et al. (2010) [55] USA	N = 294	CS	Child reported aggregate measures (encouragement, watch)	Accelerometer (7 d)	Adolescents’ MVPA & parental support: r = .15, p < .05	.19
	149 m, 145 f		α = .76	.80*		
	15.4 yrs					
Pfeiffer et al. (2009) [57] USA	N = 331	CS	Parent report	Accelerometer (8–10 d)	Children’s’ MVPA & family support: r = .04	.05
	169 m, 162 f		Parental support – aggregated measure (encouragement, co-activity, transportation, watch, inform)	.80*		
			.81 (94% respondents mothers)			
	4.3 yrs					
Prochaska et al. (2002) [81] USA	N = 138	CS	Child report	Activity monitor (5 d)	Children’s PA (monitor) & parental support: r = .12	.15
	48 m, 90 f		Parental support – aggregated & individual measures: Encouragement, coactivity, transportation, watch, praise	.70*		
	12.1 yrs					
			ICC = .88			
			α = .77			
Schaben et al. (2006) [82] USA	N = 1995	CS	Child report	Child self-report	Adolescents’ MVPA (middle school) & parental support: r = .31	.44
	1033 m, 962 f		Parental support –aggregated measure (role modeling, encouragement, co-activity, facilitation)	PAQ	Adolescents’ MVPA (high school) & parental support: r = .38	
	14.7 yrs		α = 81	Test-retest males	**Mean r = .35**	
				.75		
				Test-retest females .82		
Schary et al. (2012) [83] USA	N = 195	CS	Parent report	Parent report	Children’s PA & parental support: r = .32, p < .001	.44
			Parental support – aggregated & individual measures (encouragement, transportation, co-activity, watch, inform)	PAEC-Q		
	90 m, 105 f					
	4.0 yrs		α = .76 (86% respondents mothers)	.70*		
Taylor et al., (2002) [84] USA	N = 509	CS	Child report	Child self-report & parent report	Children’s PA & family support: partial r = .43, p < .001 (<85^th^ percentile BMI); partial r = .13, p = .45 (>85^th^ percentile BMI)	.48
	231 m, 278 f		Family support – aggregated measure (encouragement, co-activity, transportation, watch)	.70*		
	Age range: 12–18 yrs		α = .81		**Mean r = .38**	
			Test-retest .88			
Trost et al. (2003) [64] USA	N = 380	CS	Parent report	Child self-report	Adolescents’ PA & parental support: β = .24	.30
	171 m, 209 f		Parental support – aggregated measure (encouragement, co-activity, transportation, watch, inform)	Test-retest .79		
	14.0 yrs		α = .78			
			Test-retest .81			
Verloigne et al. (2014) [85] Australia	N = 134	CS	Parent report	Accelerometer (4 d)	Adolescent MVPA & logistic support: r = .02	.03
	66 m, 68 f		Parental support (logistic support – transportation, financial support)	.70*		
	14.1 yrs		α = .90 (84% respondents mothers)			
Welk et al. (2003) [66] USA	N = 994	CS	Child report	Child self-report	Children’s PA & parental support: r = .51	.70
	505 m, 489 f		Parental support – aggregated measure (encouragement, involvement, facilitation)	PAQ-C		
	10.0 yrs		α = .76	.70*		
Williams & Mummery (2011) [67] Australia	N = 295	CS	Parent report	Adolescent PARQ	Adolescents’ MVPA & parental support: OR = 7.38, 95% CI 2.98-18.29*	.95
	111 m, 184 f		Parental support – aggregated measure (encouragement, watch, transportation, inform, co-activity)	.70*	*adjusted for other variables	
	15.1 yrs		.70* (67% respondents mothers)			
Zecevic et al. (2010) [68] Canada	N = 102	CS	Parent report	Parental report	Children’s PA & parental support: OR = 2.18, p < .10; r = .2976	.41
	54 m, 48 f		Parental support – aggregated measure (encouragement, co-activity, transportation, watch, inform)	.70*		
	3.8 yrs					
			α = .75			
Zhang et al. (2012) [86] USA	N = 285	CS	Child report	PAQ-C	Adolescent’s MVPA & parental support: r = .43, p < .01	.55
	142 m, 143 f		Parental support – aggregated measure (encouragement, coactivity, transportation, praise)	.75		
	13.4 yrs		.81			

Note. *reliability not reported; CLASS = Children’s Leisure Activities Study Survey; CS = cross-sectional; d = day; f = female; GLTEQ = Godin Leisure-Time Exercise Questionnaire; m = males; MVPA = moderate to vigorous physical activity; PA = physical activity; PAQ = Physical Activity Questionnaire; PAQ-C = Physical Activity Questionnaire for Older Children; PARQ = Physical Activity Recall Questionnaire; PEACH = Personal and Environmental Associations with Children’s Health; PAEC-Q = Physical Activity and Exercise Questionnaire for Children; PRO = prospective; OSRAC-P = Observational System for Recording Physical Activity in Children-Preschool Version; wk = week.

**Table 4:** **Studies and effect sizes for parental modeling and girls' physical activity moderated by parental gender (k = 62)**

**Study, country**	**Sample (number, gender, mean age)**	**Design**	**Parental PA measure**	**Child PA measure**	**Results**	**Corrected effect size**
Aarnio et al. (1997) [87] Finland	N = 3254	CS	Parent self-report	Child self-report	Girls’ PA (n = 1130) & fathers’ PA: r = .046, p < .01	.07
	1557 m, 1697 f		.70*	.70*	Girls’ PA (n = 1123) & mothers’ PA: r = .101, p < .01	.14
	16.0 yrs					
Anderssen & Wold (1992) [88] Norway	N = 904	CS	Child report	Child self-report	Girls’ PA & fathers’ PA: r = .14, p < .01	.19
	498 m, 406 f		.70*	.78	Girls’ PA & mothers’ PA: r = .14, p < .01	.19
	13.3 yrs					
Anderssen et al. (2006) [89] Norway	N = 380	PRO (8 yrs)	Parent self-report	Child self-report	Girls’ PA & fathers’ PA: β = .09	.12
	191 m, 189 f		.70*	.83	Girls’ PA & mothers’ PA: β = .05	.07
	13.3 yrs at baseline					
Bastos et al. (2008) [90] Brazil	N = 857	CS	Child report	Child self-report	Girls’ PA & fathers’ PA: r = −.08	-.11
	411 m, 446 f		.70*	.70*	Girls’ PA & mothers’ PA: r = −.05	-.07
	Age range: 10–19 yrs					
Bogaert et al. (2003) [91] Australia	N = 59	PRO (1 yr)	Parent self-report	Parent report	Girls’ MVPA & mothers PA: r = .44, p = .03	.47
	29 m, 30 f		Bouchard activity record .97	Bouchard activity record .91		
	8.6 yrs at baseline					
Campbell et al. (2001) [92] Canada	N = 153	PRO (12 yrs)	Parent self-report	Child self-report	Girls’ MVPA & fathers’ PA: r = .001	.00
	77 m, 76 f		.97	.91	Girls’ MVPA & mothers’ PA: r = .008	.01
	13.5 yrs at baseline					
Davison et al. (2001) [93] USA	N = 197	PRO	Parent self-report	Parent report	Girls’ PA & fathers’ PA: r = −.03 at baseline	-.05
	All females	(2 yrs)	.70*	α = .58	Girls’ PA & mothers’ PA: r = .09 at baseline	.14
	5.4 yrs at baseline					
	7.3 at follow-up					
Davison et al. (2003) [94] USA	N = 180	CS	Parent report	PA measures as a composite score of CPA-short, an activity checklist, and PACER	Girls’ & fathers’ explicit modeling: r = .25, p < .01	.36
	All females		Explicit modeling	.70*	Girls’ PA and mothers’ explicit modeling: r = .08	.11
	9.0 yrs		.70*			
Deflandre et al. (2001) [95] France	N = 80	CS	Child report	Child self-report	Girls’ MVPA & fathers’ PA: r = .30	.43
	36 m, 44 f		.70*	.70*	Girls’ MVPA & mothers’ PA: r = .16	.23
	Age range: 11–16 yrs					
Deflandre et al. (2001) [96] France	N = 48	CS	Child report	Heart rate monitor (7 d)	Girls’ MVPA & fathers ‘PA: r = .35	.50
	26 m, 22 f		.70*	.70*	Girls’ MVPA & mothers’ PA: r = .21	.30
	17.0 yrs					
Eriksson et al. (2008) [97] Sweden	N = 1124	CS	Baeke Questionnaire	Child self-report	Girls’ PA (sports) & fathers’ PA: crude OR = 2.2, 95% CI 1.1-4.2	.43
	553 m, 571 f		.70*	.70*	Girls’ PA (sports) & mothers’ PA: crude OR = 3.0, 95% CI 1.4-4.5	.58
	12.0 yrs					
Fogelholm et al. (1999) [98] Finland	N = 271	CS	Parent self-report	Child self-report	Girls’ VPA & fathers’ PA: r = .24, p < .01	.34
	143 m, 128 f		.70*	.70*	Girls’ VPA & mothers’ PA: r = .28, p < .01	.40
	9.6 yrs					
Fuemmeler et al. (2011) [99] USA	N = 45	CS	Accelerometer (3 d)	Accelerometer (3 d)	Girls’ MVPA (weekend) & fathers’ PA: r = .37	.47
	23 m, 22 f		.70*	.70*	Girls’ MVPA (weekday) & fathers’ PA; r = .42, p < .05; r = .19	.96
	9.9 yrs				**Mean r = .327**	
					Girls’ MVPA (weekday) & mothers’ PA: r = .70, p < .01; r = .64, p < .01	
					Girls’ MVPA (weekend) & mothers’ PA: r = .67, p < .01	
					**Mean r = .670**	
Hinkley et al. (2012) [100] Australia	N = 705	CS	Parent self-report	Accelerometer (8 d)	Girls’ PA & father’s MPA: OR = 1.01, 95% CI 1.00-1.02, p < .05	.01
	366 m, 262 f		.70* (94% respondents mothers)	.80*	Girls’ PA & mother’s VPA: OR = 1.01, 95% CI .99-1.02	.01
	4.5 yrs					
Jacobi et al. (2011) [101] France	N = 630	CS	Parent self-report	Pedometer (7 d)	Girls’ PA & mothers’ PA: r = .24	.34
	317 m, 313 f		MAQ	.70*		
	Age range 8–18 yrs		Pedometer (7 d)			
			.70*			
Jago et al. (2014) [102] UK	N = 822	CS	Accelerometer (5 d)	Accelerometer (5 d)	Girls’ MVPA & fathers’ MVPA: β = .07	.10
	436 m, 386 f		.70*	.70*	Girls’ MVPA & mothers’ MVPA: β = .15	.21
	6.0 yrs					
Kahn et al. (2008) [103] USA	N = 12812	PRO (1 yr)	Parent self-report	Child self-report	Girls’ MVPA & mothers’ PA: β = .13, p < .0001	.19
	5575 m, 7237 f		.70*	.70*		
	Age range: 10–18 yrs					
Madsen et al. (2009) [104] USA	N = 2379	PRO (9 yrs)	Parent self-report	Child self-report	Girls’ MVPA & child reported fathers’ PA (yr 3): r = .13, p < .05	.19
	All females		.70*	HAQ	Girls’ MVPA & child reported fathers’ PA (yr 5): r = .08	.20
	9-10 yrs followed to 18–19 yrs		Adolescent report	.70*	Girls’ MVPA & child reported fathers’ PA (yr 7): r = .13, p < .05	
			.70*		Girls’ MVPA & fathers’ PA (yr 9): r = .18, p < .05	
					**Mean r = .13**	
					Girls’ MVPA & child reported mothers’ PA (yr 3): r = .13, p < .05	
					Girls’ MVPA & child reported mothers’ PA (yr 5): r = .12	
					Girls’ MVPA & child reported mothers’ PA (yr 7): r = .16, p < .05	
					Girls’ MVPA & child reported mothers’ PA (yr 9): r = .16, p < .05	
					**Mean r = .14**	
Martin-Matillas et al. (2011) [105] Spain	N = 2260	CS	Child report	Child self-report	Girls’ PA & fathers’ PA: OR = 2.37, 95% CI 1.70-3.29, p < .001	.47
	1157 m, 1103 f		Health Behaviour in Schoolchildren	.70*	Girls’ PA & mothers’ PA: OR = 1.90, 95% CI 1.41-2.56, p < .001	.35
	Age range; 13–18.5 yrs		.70*			
Moore et al. (1991) [52] USA	N = 100	CS	Accelerometer (7–9 d)	Accelerometer (8–9 d)	Girls’ PA & fathers’ PA: OR = 4.4, 95% CI 1.5-8.2	.66
	63 m, 37 f		.80*	.80*	Girls’ PA & mothers’ PA: OR = 2.0, 95% CI .9-4.4	.33
	Age range: 4–7 yrs					
Nichols-English et al. (2006) [106] USA	N = 133	CS	Parent self-report	Child self-report	Girls’ MPA & mothers’ MPA: r = .05	-.09
	All female		7DPAR	7DPAR	Girls’ VPA & mothers’ VPA: r = −.16	
	9.6 yrs		.70*	.70*	**Mean r = −.06**	
O’Loughlin et al. (1999) [107] Canada	N = 1920	CS	Child report .70*	Child self-report	Girls’ PA (sports) & mothers’ PA: OR = 1.6 95% CI 1.1-2.1	.26
	989 m, 931 f			Weekly activity checklist		
	Age range: 9–13 yrs			Self-reported sports participation		
				.70*		
Ohta et al. (2010) [108] Japan	N = 339	CS	Parent self-report	Child self-report	Girls’ PA & mothers’ PA: r = .163, p < .01	.23
	All female		.70*	.70*		
	14.8 yrs					
Pahkala et al. (2007) [109] Finland	N = 558	CS	Parent self-report	Child self-report	Girls’ PA & fathers’ PA: r = .10, p = .19	.21
	294 m, 264 f		.70*	.70*	Girls’ PA & mothers’ PA: r = .15, p < .05	.17
	13.0 yrs					
Raudsepp (2006) [110] Estonia	N = 329	CS	Parent self-report	Child self-report	Girls’ MVPA & fathers’ explicit modeling: r = .23, p < .01	.32
	168 m, 161 f		Father’s modeling .72	7DPAR	Girls’ MVPA & mothers’ explicit modeling: r = .33, p < .01	.47
	13.8 yrs					
			Mother’s modeling .71	.70*		
Shropshire & Carroll (1997) [111] UK	N = 924	CS	Child report	Child self-report	Girls’ PA & fathers’ PA: r = .23	.33
	468 m, 454 f		.70*	.70*	Girls’ PA & mothers’ PA: r = .05	.07
	Age range: 10–11 yrs					
Siegel et al. (2011) [112] Mexico	N = 1004	CS	Child report	Child self-report PAQ	Girls’ MVPA (9–10 yrs) & fathers’ PA: β = .186, p < .05	.26
	490 m, 514 f		.70*	α = .72	Girls’ MVPA (9–10 yrs) & mothers’ PA: β = .148, p < .05	.21
	Age range: 9–18 yr olds					
					Girls’ MVPA (11–13 yrs) & fathers’ PA: β = .151, p < .05	.21
					Girls’ MVPA (11–13 yrs) & mothers’ PA: β = .191, p < .05	.27
Sigmund et al. (2008) [113] Czech Republic	N = 192	CS	Parent report	Child report	Girls’ MPA & fathers’ PA: r = .15	.19
	109 m, 89 f		IPAQ	IPAQ	Girls’ VPA & fathers’ PA: r = .10	.20
	Age range: 8–13 yrs		.70*	.70*	**Mean r = .13**	
					Girls’ MPA & mothers’ PA: r = .28	
					Girls’ VPA & mothers’ PA: r = .27	
					**Mean r = .14**	
Toftegaard-Stockel et al. (2011) [114] Denmark	N = 6356	CS	Child report	Child self-report	Girls’ PA (sports) & fathers’ PA: r = .11	.16
	3190 m, 3166 f		.70*	.70*	Girls’ PA (sports) & mothers’ PA: r = .22	.31
	Age range: 12–16 yrs					
Trost et al. (1997) [115] USA	N = 202	PRO	Child report	Child report	Girls’ MVPA & fathers’ PA: r = −.02	-.02
	92 m, 110 f	(1 yr)	.70*	PDPAR	Girls’ MVPA & mothers’ PA: r = .09	.11
	10-11 yrs at baseline			.98		
Trost et al. (1999) [116] USA	N = 198	CS	Child report	Accelerometer (7 d)	Girls’ VPA & fathers’ PA: r = .10	.16
	95 m, 103 f		.70*	.80*	Girls’ MPA & fathers’ PA: r = .13	.12
	11.4 yrs				**Mean r = .12**	
					Girls’ VPA & mothers’ PA: r = .08	
					Girls’ MPA & mothers’ PA: r = .09	
					**Mean r = .09**	
Wagner et al. (2004) [117] France	N = 2852	CS	Parent self-report	Child self-report	Girls’ structured PA & fathers’ PA: OR = 1.41, 95% CI 1.03-1.92	.25
	1421 m, 1431 f		.70*	MAQ-A	Girls’ structured PA & mothers’ PA: OR = 1.80, 95% CI 1.28-2.52	.19
	12.0 yrs			.70*		
Yang et al. (1996) [118] Finland	N = 635	PRO (12 yrs)	Child report	Child self-report	Girls’ PA (cohort 1) & fathers’ PA: r = .12	.20
	316 m, 319 f		.70*	.70*	Girls’ PA (cohort 2) & fathers’ PA: r = .14	.17
	9.0 yrs at baseline				Girls’ PA (cohort 3) & fathers’ PA: r = .15	
	N = 648				**Mean r = .14**	
	321 m, 327 f				Girls’ PA (cohort 1) & mothers’ PA: r = .14	
	12.0 yrs at baseline				Girls’ PA (cohort 2) & mothers’ PA: r = .12	
	N = 598				Girls’ PA (cohort 3) & mothers’ PA: r = .12	
	286 m, 312 f				**Mean r = .12**	
	15.0 yrs at baseline					

*Note.* *reliability not reported; 7DPAR = 7-Day Physical Activity Recall; CPA = Children’s Physical Activity-Short Scale; CS = cross-sectional; d = day; f = female; HAQ = Habitual Activity Questionnaire; IPAQ = International Physical Activity Questionnaire; m = male; MAQ = Modifiable Activity Questionnaire; MAQ-A = Modifiable Activity Questionnaire for Adolescents; MPA = moderate physical activity; MVPA = moderate to vigorous physical activity; PA = physical activity; PACER = Progressive Aerobic Cardiovascular Endurance Run; PAQ = Physical Activity Questionnaire; PDPAR = Previous Day Physical Activity Recall; PRO = prospective; VPA = vigorous physical activity.

**Table 5:** **Studies and effect sizes for parental modeling and boys' physical activity moderated by parental gender (k = 49)**

**Study, country**	**Sample (number, gender, mean age)**	**Design**	**Parental PA measure**	**Child PA measure**	**Results**	**Corrected effect size**
Aarnio et al. (1997) [87] Finland	N = 3254	CS	Parent self-report	Child self-report	Boys’ PA (n = 1120) & fathers’ PA: r = .012, p < .01	.02
	1557 m, 1697 f		.70*	.70*	Boys’ PA (n = 1146) & mothers’ PA: r = .100, p < .01	.14
	16.0 yrs					
Anderssen & Wold (1992) [88] Norway	N = 904	CS	Child report	Child self-report	Boys’ PA & fathers’ PA: r = .17, p < .001	.23
	498 m, 406 f		.70*	.78	Boys’ PA & mothers’ PA: r = .11, p < .01	.15
	13.3 yrs					
Anderssen et al. (2006) [89] Norway	N = 380	PRO (8 yrs)	Parent self-report	Child self-report	Boys’ PA & fathers’ PA: β = .10	.13
	191 m, 189 f		.70*	.83	Boys’ PA & mothers’ PA: β = .11	.14
	13.3 yrs at baseline					
Bastos et al. (2008) [90] Brazil	N = 857	CS	Child report	Child self-report	Boys’ PA & fathers’ PA: r = −.02	-.03
	411 m, 446 f		.70*	.70*	Boys’ & mothers’ PA: r = .08	.11
	Age range: 10–19 yrs					
Campbell et al. (2001) [92] Canada	N = 153	PRO (12 yrs)	Parent self-report	Child self-report	Boys’ MVPA & fathers’ PA: r = .05	.05
	77 m, 76 f		.97	.91	Boys’ MVPA & mothers’ PA: r = .03	.03
	13.5 yrs at baseline					
Deflandre et al. (2001) [95] France	N = 80	CS	Child report	Child self-report	Boys’ MVPA & fathers’ PA: r = .56	.80
	36 m, 44 f		.70*	.70*	Boys’ MVPA & mothers’ PA: r = .30	.43
	Age range: 11–16 yrs					
Deflandre et al. (2001) [96] France	N = 48	CS	Child report	Heart rate monitor (7 d)	Boys’ MVPA & fathers’ PA: r = −.11	-.16
	26 m, 22 f		.70*	.70*	Boys’ MVPA & mothers’ PA: r = −.01	-.01
	17.0 yrs					
Eriksson et al. (2008) [97] Sweden	N = 1124	CS	Baeke Questionnaire	Child self-report	Boys’ PA (sports) & fathers’ PA: crude OR = 3.2, 95% CI 1.5-6.6	.61
	553 m, 571 f		.70*	.70*	Boys’ PA (sports) & mothers’ PA: crude OR = 2.5, 95% CI 1.4-4.5	.49
	12.0 yrs					
					crude OR = 3.0, 95% CI 1.4-4.5	
Fogelholm et al. (1999) [98] Finland	N = 271	CS	Parent self-report	Child self-report	Boys’ VPA & fathers’ PA: r = .08	.11
	143 m, 128 f		.70*	.70*	Boys’ VPA & mothers’ PA: r = .20, p < .01	.29
	9.6 yrs					
Fuemmeler et al. (2011) [99] USA	N = 45	CS	Accelerometer (3 d)	Accelerometer (3 d)	Boys’ MVPA (weekend) & fathers’ PA: r = .43, p < .05	.65
	23 m, 22 f		.70*	.70*	Boys’ MVPA (weekday) & fathers’ PA: r = .38; r = .55, p < .01	.15
	9.9 yrs				**Mean r = .453**	
					Boys’ MVPA (weekend) & mothers’ PA: r = .10	
					Boys’ MVPA (weekday) & mothers’ PA: r = .09; r = .13	
					**Mean r = .107**	
Jacobi et al. (2011) [101] France	N = 630	CS	Parent self-report	Pedometer (7 d)	Boys’ PA & mothers’ PA: r = .18	.26
	317 m, 313 f		MAQ	.70*		
	Age range 8–18 yrs		Pedometer (7 d)			
			.70*			
Jago et al. (2014) [102] UK	N = 822	CS	Accelerometer (5 d)	Accelerometer (5 d)	Boys’ MVPA & fathers’ MVPA: β = .10	.14
	436 m, 386 f		.70*	.70*	Boys’ MVPA & mothers’ MVPA: β = .06	.09
	6.0 yrs					
Kahn et al. (2008) [103] USA	N = 12812	PRO (1 yr)	Parent self-report	Child self-report	Boys’ MVPA & mothers’ PA: β = .085, p < .0001	.12
	5575 m, 7237 f		.70*	.70*		
	Age range: 10–18 yrs					
Martin-Matillas et al. (2011) [105] Spain	N = 2260	CS	Child report	Child self-report	Boys’ PA & fathers’ PA: OR = 1.99, 95% CI 1.40-2.84, p < .001	.38
	1157 m, 1103 f		Health Behaviour in School Children	.70*		.09
	Age range; 13–18.5 yrs		.70*		Boys’ PA & mothers’ PA: OR = 1.18, 95% CI .85-1.65, p > .05	
Moore et al. (1991) [52] USA	N = 100	CS	Accelerometer (7–9 d)	Accelerometer (8–9 d)	Boys’ PA & fathers’ PA: OR = 3.1, 95% CI 1.1-9.3	.52
	63 m, 37 f		80*	.80*	Boys’ PA & mothers’ PA: OR = 2.0, 95% CI .7-5.7	.33
	Age range: 4–7 yrs					
O’Loughlin et al. (1999) [107] Canada	N = 1920	CS	Child report .70*	Child self-report	Boys’ PA (sports) & fathers’ PA: OR = 2.0, 95% CI 1.4-2.9	.38
	989 m, 931 f			Weekly Activity Checklist		
	Age range: 9–13 yrs			Self-reported sports participation		
				.70*		
Pahkala et al. (2007) [109] Finland	N = 558	CS	Parent self-report	Child self-report	Boys’ PA & fathers’ PA: r = .07, p = .28	.10
	294 m, 264 f		.70*	.70*	Boys’ PA & mothers’ PA: r = .10, p = .13	.14
	13.0 yrs					
Raudsepp (2006) [110] Estonia	N = 329	CS	Parent self-report	Child self-report	Boys’ MVPA & fathers’ explicit modeling: r = .38, p < .001	.54
	168 m, 161 f		Father’s modeling .72	7DPAR	Boys’ MVPA & mothers’ explicit modeling: r = .35, p < .01	.50
	13.8 yrs		Mother’s modeling .71	.70*		
Shropshire & Carroll (1997) [111] UK	N = 924	CS	Child report	Child self-report	Boys’ PA & fathers’ PA: r = .19	.27
	468 m, 454 f		.70*	.70*	Boys’ PA & mothers’ PA: r = .11	.16
	Age range: 10–11 yrs					
Siegel et al. (2011) [112] Mexico	N = 1004	CS	Child report	Child self-report PAQ	Boys’ MVPA (9–10 yrs) & fathers’ PA: β = .239, p < .05	.34
	490 m, 514 f		.70*	α = .72	Boys’ MVPA (9–10 yrs) & mothers’ PA: β = .160, p < .05	.23
	Age range: 9–18 yr olds					
Sigmund et al. (2008) [113] Czech Republic	N = 192	CS	Parent report	Child report	Boys’ MPA & fathers’ PA: r = .39, p < .001	.34
	109 m, 89 f		IPAQ	IPAQ	Boys’ VPA & fathers’ PA: r = .08	.34
	Age range: 8–13 yrs		.70*	.70*	**Mean r = .24**	
					Boys’ MPA & mothers’ PA: r = .30	
					Boys’ VPA & mothers’ PA: r = .17	
					**Mean r = .24**	
Toftegaard-Stockel et al. (2011) [114] Denmark	N = 6356	CS	Child report	Child self-report	Boys’ PA (sports) & fathers’ PA: r = .19	.27
	3190 m, 3166 f		.70*	.70*	Boys’ PA (sports) & mothers’ PA: r = .06	.09
	Age range: 12–16 yrs					
Trost et al. (1997) [115] USA	N = 202	PRO (1 yr)	Child report	Child report	Boys’ MVPA & fathers’ PA: r = .05	.06
	92 m, 110 f		.70*	PDPAR	Boys’ MVPA & mothers’ PA: r = −.07	-.08
	10-11 yrs at baseline			.98		
Trost et al. (1999) [116] USA	N = 198	CS	Child report	Accelerometer (7 d)	Boys’ VPA & fathers’ PA: r = .15	.24
	95 m, 103 f		.70*	.80*	Boys’ MPA & fathers’ PA: r = .21, p < .05	.24
	11.4 yrs				**Mean r = .18**	
					Boys’ VPA & mothers’ PA: r = .21, p < .05	
					Boys’ MPA & mothers’ PA: r = .14	
					**Mean r = .18**	
Wagner et al. (2004) [117] France	N = 2852	CS	Parent self-report	Child self-report	Boys’ structured PA & fathers’ PA: OR = 1.36, 95% CI .97-1.91	.37
	1421 m, 1431 f		.70*	MAQ-A	Boys’ structured PA & mothers’ PA: OR = 1.48, 95% CI 1.01-2.14	.17
	12 yrs			.70*		
Yang et al. (1996) [118] Finland	N = 635	PRO (12 yrs)	Child report	Child self-report	Boys’ PA (cohort 1) & fathers’ PA: r = .21	.27
	316 m, 319 f		.70*	.70*	Boys’ PA (cohort 2) & fathers’ PA: r = .17	.10
	9 yrs at baseline				Boys’ PA (cohort 3) & fathers’ PA: r = .18	
	N = 648				**Mean r = .19**	
	321 m, 327 f				Boys’ PA (cohort 1) & mothers’ PA: r = .08	
	12 yrs at baseline				Boys’ PA (cohort 2) & mothers’ PA: r = .06	
					Boys’ PA (cohort 3) & mothers’ PA: r = .08	
	N = 598				**Mean r = .07**	
	286 m, 312 f					
	15 yrs at baseline					

*Note.* *reliability not reported; 7DPAR = 7-Day Physical Activity Recall; CS = cross-sectional; d = days; f = females; IPAQ = International Physical Activity Questionnaire; m = males; MAQ = Modifiable Activity Questionnaire; MAQ-A = Modifiable Activity Questionnaire for Adolescents; MPA = moderate physical activity; MVPA = moderate to vigorous physical activity; PA = physical activity; PAQ = Physical Activity Questionnaire; PDPAR = Previous Day Physical Activity Recall; PRO = prospective; VPA = vigorous physical activity.

**Table 6:** **Studies and effect sizes for individual parental support behaviours (k = 64)**

**Study, country**	**Sample (number, gender, mean age)**	**Design**	**Parental support measure**	**Child physical activity measure**	**Results**	**Corrected effect size**
Anderson et al. (2007) [119] USA	N = 100	CS	Child report	Accelerometer (4 d)	Children’s MPA & encouragement: r = −.06	.05
	47 m, 53 f		Encouragement	.70*	Children’s VPA & encouragement: r = .11	
	13.4 yrs		.63		**Mean r = .03**	
Anderson et al. (2009) [120] USA	N = 391	CS	Child report	Child self-report	Children’s MVPA & encouragement: r = .39	.56
	207 m, 184 f		Encouragement	PAQ-C	Adolescents’ MVPA & parental encouragement: r = .25	.36
	9.9 yrs		.70*	.70*		
	N = 948			Child self-report		
	370 m, 578 f			MAQ-A		
	13.6 yrs			.70*		
Arredondo et al. (2006) [121] USA	N = 812	CS	Parent report	Parent report	Children’s PA & monitoring: β = .19, p < .001*	.27
	390 m, 422 f		Monitoring	.70*	Children’s PA & praise: β = .13, p < .001*	.19
			.70*		*Adjusted for parent’s age, marital status, employment, & education	
	6.0 yrs					
			Reinforcement/praise			
			.70*			
Beets et al. (2006) [122] USA	N = 363	CS	Child report	Child self-report	Children’s MVPA & providing transportation: β = .28	.40
	174 m, 189 f		Encouragement	Youth risk behavior surveillance survey	Children’s MVPA & praise: β = .36	.51
	12.3 yrs		.70*	.70*		
			Transportation			
			.70*			
			Co-activity			
			.70*			
			Watch.			
			70*			
			Praise			
			.70*			
De Bourdeaudhuij et al. (2005) [123] Belguim	N = 5563 (normal weight)	CS	Child report	Child self-report	Children’s PA & encouragement: r = .25 (normal weight); r = .26 (overweight)	.35
	14.8 yrs		Encouragement	Study developed questionnaire	**Mean r = .25**	
	N = 515 (overweight & obese)		.70*	.75		
	14.6 yrs					
Dowda et al. (2011) [40] USA	N = 369	CS	Parent report	Accelerometer (2 wk)	Children’s MVPA & PA equipment: β = .17	.22
	175 m, 194 f		PA equipment at home	.80*		
	4.2 yrs		.70* (92% respondents mothers)	Direct observation (OSRAC-P)		
				Inter-observer .91		
Fredricks & Eccles (2005) [42] USA	N = 364	PRO (1 yr)	Parent report	Child self-report	Children’s PA (sport) & encouragement: r = .33, p < .001 (baseline); r = .31, p < .001 (follow-up)	.45
	184 m, 180 f		Encouragement & co-activity	.70*	**Mean r = .32**	.08
	Ages 7.0-11.0 yrs at baseline					
			α = .73		Children’s PA (sport) & co-activity: r = .05 (baseline); r = .07 (follow-up)	.36
			Equipment purchases		**Mean r = .06**	
					Children’s PA (sport) & PA equipment: r = .24, p < .001 (baseline); r = .25, p < .001	
			.70*		**Mean r = .25**	
Gubbels et al. (2011) [124] Netherlands	N = 2026	PRO (2 yrs)	Parent report	Parent report	Children’s PA & encouragement: β = .06, p < .05	.09
	1037 m, 989 f		Encouragement	.70*		
	5.0 yrs at baseline		α = .57			
Heitzler et al. (2006) [76] USA	N = 3114	CS	Parent reported	Child self-report	Children’s organized PA & transportation: OR = 1.21, 95% CI 1.11-1.33, p < .001	.12
	Age range: 9.0-13.0 yrs		Parental support – aggregated and individually reported (co-activity, watching child, & transportation)	Test-retest .64	Children’s organized PA & watching: OR = 1.31, 95% 1.19-1.43, p < .001	.16
			Test-retest .65		Children’s organized PA & co-activity: OR = 1.08, 95% CI 1.02-1.13, p < .001	.01
Hendrie et al. (2011) [44] Australia	N = 106	CS	Parent report	Child self-report	Children’s organized PA & co-activity: r = .247, p < .05; r = .286, p < .01 when controlled for parent demographic factors	.33
	51 m, 55 f		Parental support – aggregate measure (watch, transportation)	CLASS		
	8.3 yrs		α = .79	.70*		
			Co-activity			
			α = .79			
			(92% respondents mothers)			
Hennessy et al. (2010) [45] USA	N = 76	CS	Parent report	Accelerometer (5 d)	Children’s PA & monitoring: β = −.13	-.17
	26 m, 50 f		Monitoring	.70*	Children’s PA & praise: β = −.05, p = .68	-.07
	9.1 yrs		Reinforcement			
			.83			
Hohepa et al. (2007) [125] New Zealand	N = 3471	CS	Child report	Child report	Adolescents’ PA & encouragement: r = .38 (juniors); r = .41 (seniors)	.56
	1666 m, 1805 f		Encouragement	.70*	**Mean r = .39**	
	14.8 yrs		.70*			
Huang et al. (2011) [126] China	N = 303	CS	Child report	Child self-report	Children’s MVPA & PA equipment: r = .14, p < .05	.20
	143 m, 160 f		Availability of PA equipment	CLASS-C		
	11.1 yrs		.70*	.70*		
Klesges et al. (1984) [127] USA	N = 14	CS	Direct observation (FATS)	Direct observation (FATS)	Children’s PA (activity monitor) & encouragement: r = .23	.29
	7 m, 7 f		Encouragement	.90		
	2.8 yrs		.90	Activity monitor		
				.70*		
Klesges et al. (1986) [128] USA	N = 30	CS	Direct observation (FATS)	Direct observation (FATS)	Children’s PA & encouragement: r = .32, p < .05	.36
	15 m, 15 f		Encouragement	.90		
	2.5 yrs		.90			
Klesges et al. (1990) [129] USA	N = 222	CS	Direct observation (CATS)	Direct observation (CATS)	Children’s PA & encouragement: β = .32, p = .648	.35
	122 m, 100 f		Encouragement	.91		
	4.4 yrs		.91			
King et al. (2008) [130] USA	N = 535	CS	Child report	Child self-report	Children’s VPA & encouragement: r = .15	.20
	290 m, 245 f		Encouragement	.70*	Children’s MPA & encouragement: r = .13	
	Age range: 14–18 yrs		.70*			
					**Mean r = .14**	
Lawman & Wilson (2014) [79] USA	N = 181	CS	Parent report	Accelerometer (7 d)	Adolescent’s MVPA & PA equipment: r = .09	.13
	72 m, 109 f		Availability of PA equipment	.80*	Adolescent’s MVPA & monitoring: r = .07	.08
	13.3 yrs		α = .61			
			Monitoring			
			α = .86			
Loprinzi et al. (2013) [50] USA	N = 176	CS	Parent report	Parent report	Children’s MVPA & monitoring: β = .20, p < .05	.29
	82 m, 94 f		Monitoring child’s PA	PAEC-Q		
	4.0 yrs		.70 (85% respondents mothers)	.70*		
Loucaides et al. (2004) [131] Cyprus	N = 256	CS	Child report	Child self-report	Children’s MVPA & encouragement: r = .12 (winter); r = .13 (summer)	.10
	Age range: 11.0-12.0 yrs		Encouragement	PDPAR	**Mean r = .08**	
			Test-test .64	Test-retest .96	Children’s MVPA & PA equipment: r = .25, p < .001 (winter); r = .18, p < .01 (summer)	
			Parent report		**Mean r = .22**	.27
			Availability of PA equipment			
			.70*			
Loucaides & Jago (2006) [132] Cyprus	N = 104	CS	Parent report	Pedometer (5 d)	Children’s PA & equipment: r = .10	.14
	54 m, 50 f		PA equipment	.70*	Children’s PA & transportation: r = .17	.24
	Age range: 10.0-12.0 yrs		.70*		Children’s PA & watching: r = .18	.22
			Transportation			
			.70*			
			Accompany child to PA			
			.99			
Määtä et al. (2014) [133] Finland	N = 883	CS	Child report	Child self-report	Child PA & encouragement: r = .19, p < .001	.25
	Age range: 10–11 yrs		Encouragement	.70*	Child PA & Co-activity: r = .16, p < .001	.24
			α = .84			
			Co-activity			
			α = .63			
McKenzie et al. (1991) [134] USA	N = 42	PRO	Direct observation (BEACHES)	Direct observation (BEACHES)	Children’s PA & encouragement: r = .43, p < .01	.51
	17 m, 25 f	(8 wks)	Prompts to be active	.85		
	Age range: 4.0-8.0 yrs		.85			
McKenzie et al. (2008) [135] USA	N = 139	CS	Direct observation (BEACHES)	Direct observation (BEACHES)	Children’s MVPA & encouragement: r = .53, p < .01	.62
	69 m, 70 f		Prompts to be active	.85		
	6.5 yrs		.85 (97% respondents mothers)			
Millstein et al. (2011) [136] USA	N = 104	CS	Parent report	Parent report	Children’s MVPA & PA equipment at home: r = .14, p < .15	.18
	8.3 yrs		Availability of PA equipment	ICC = .76	Children’s MVPA & providing recreation centre membership: r = .04	.05
	N = 137		ICC = .80	Child self-report	Adolescents’ MVPA & PA equipment at home: r = .28, p < .01	.42
	14.6 yrs		Provision of recreation centre membership	ICC = .64	Adolescent’s MVPA & providing recreation centre membership: r = .24, p < .01	.37
			ICC = .76			
			Child report			
			Availability of PA equipment			
			ICC = .69			
			Provision of recreation centre membership			
			ICC = .66			
Moore et al. (2008) [137] USA	N =116	CS	Child report	Child self-report	Adolescents’ MVPA & financial support (lessons): OR = 2.79, 95% CI 1.18-6.60, p < .05	.50
	46 m, 70 f		Financial support	.70*	Adolescents’ MVPA & financial support (sports): OR = 5.61, 95% CI 2.30-13.70, p < .01	
	Age range: 9.0-17.0 yrs		.70*		**Mean r = .35**	
Moore et al. (2014) [138] USA	N = 1005	CS	Child report	Accelerometer (4 d)	Children’s MVPA & watching: OR .99, 95% CI .87-1.14); r = −.004	-.01
	11.3 yrs		Watch, praise, transportation, co-activity	.70*	Children’s MVPA & praise: OR 1.01, 95% CI .88-1.12; r = .004	.01
			α = .76-.90		Children’s MVPA & transportation: OR 1.28, 95% CI 1.12-1.45, p < .05; r = .14	.19
					Children’s MVPA & co-activity: OR .99, 95% CI .87-1.12; r = −.004	-.01
Mota (1998) [53] Portugal	N = 45	CS	Parent self-report	Child self-report	Children’s PA & co-activity: r = .35, p < .05	.44
	18 m, 27 f		.88	.72		
	10.1 yrs					
Nelson et al. (2005) [139] USA	N = 1681	PRO (8 yrs)	Co-activity	7DPAR	Children’s MVPA & co-activity: OR = 5.84, 95% CI 5.02-6.80	.87
	14.9 yrs at baseline		.70*	.70*		
Østbyte et al. (2013) [54] USA	N = 208	CS	Parent report	Accelerometer (7 d)	Children’s MVPA & PA equipment at home: r = .01	.01
	116 m, 92 f		PA equipment	.80*		
	2-5 yrs		α = .65 (all mothers)			
Pate et al. (1997) [140] USA	N = 361	CS	Child report	Child self-report	Children’s MVPA & PA equipment: r = .10	.13
	176 m, 185 f		PA equipment at home	PDPAR		
	10.7 yrs		ICC = .86	.70*		
Patnode et al. (2010) [55] USA	N = 294	CS	Parent reported the provision of PA equipment	Accelerometer (7 d)	Adolescents’ MVPA & PA equipment: r = .21, p < .001	.27
	149 m, 145 f		.70*	.80*		
	15.4 yrs					
Pfeiffer et al. (2009) [57] USA	N = 331	CS	Providing PA equipment	Accelerometer (8–10 d)	Children’s’ MVPA & PA equipment: r = .10, p < .10	.13
	169 m, 162 f		.70* (94% respondents mothers)			
	4.3 yrs			.80*		
Prochaska et al. (2002) [81] USA	N = 138	CS	Child report	Activity monitor (5 d)	Children’s PA (monitor) & encouragement: r = .07	.09
	48 m, 90 f		Parental support – aggregated & individual measures: Encouragement, coactivity, transportation, watch, praise	.70*	Children’s PA (monitor) & coactivity: r = .08	.10
					Children’s PA (monitor) & transportation: r = .02	.03
	12.1 yrs		ICC = .88	ICC = .79	Children’s PA (monitor) & watching: r = .16	.20
			α = .77		Children’s PA (monitor) & praise: r = .12 .30, p < .01	.15
Sabiston & Crocker (2008) [141] Canada	N = 857	CS	Child report	GLTEQ	Adolescents’ MVPA & encouragement: r = .27, p < .05	.37
	419 m, 438 f		Encouragement .77	.70*		
	16.3 yrs					
Schary et al. (2012) [84] USA	N = 195	CS	Parent report	Parent report	Children’s PA & encouragement: r = .28, p < .001	.38
	90 m, 105 f		Parental support – aggregated & individual measures (encouragement, transportation, co-activity, watch, inform)	PAEC-Q	Children’s PA & watching: r = .22	.30
	4.0 yrs		α = .76 (86% respondents mothers)	.70*	Children’s PA & co-activity: r = .26, p < .001	.36
					Children’s PA & transportation: r = .22, p < .001	.30
					Children’s PA & providing information: r = .16, p < .05	.22
Vella et al. (2014) [65] Australia	N = 4042	CS	Parent report	Parent report	Children’s PA & co-activity: OR = 1.40, 95% CI 1.24-1.57 p < .05; r = .13	.19
	2069 m, 1973 f		Co-activity	Organized sports participation		
			.70* (96% respondents mothers)	.70*		
	8.3 yrs					
Verloigne et al. (2014) [85] Australia	N = 134	CS	Parent report	Accelerometer (4 d)	Adolescent MVPA & co-activity: r = .01	.01
	66 m, 68 f		Co-activity	.70*	Adolescent MVPA & praise: r = .01	.01
	14.1 yrs		Praise			
			.70* (84% respondents mothers)			
Zhao & Settles (2014) [69] USA	N = 1514	CS	Parent self-report	Parent report	Children’s MPA & encouragement: β = .30, p < .05	.24
	763 m, 751 f		Encouragement	.70*	Children’s VPA & encouragement: β = .14	.33
	11.8 yrs		Co-activity		**Mean β = .17**	
			.70*		Children’s MPA & co-activity: β = .21, p < .01	
					Children’s VPA & co-activity: β = .25, p < .01	
					**Mean β = .23**	

*Note.* *reliability not reported; 7DPAR = 7-Day Physical Activity Recall; BEACHES = Behaviors of Eating and Activity for Children’s Health Evaluation System; CS = cross-sectional; d = days; f = females; FATS = Fargo Activity Timesampling Survey; GLTEQ = Godin Leisure-Time Exercise Questionnaire; m = males; MPA = moderate physical activity; MVPA = moderate to vigorous physical activity; PA = physical activity; PAEC-Q = Physical Activity and Exercise Questionnaire for Children; PAQ-C = Physical Activity Questionnaire for Older Children; PRO = prospective; CATS = Children’s Activity Timesampling Survey; VPA = vigorous physical activity.

**Table 7:** **Studies and effect sizes for parental support and girls’ physical activity moderated by parental gender (k = 13)**

**Study, country**	**Sample (number, gender, mean age)**	**Design**	**Parental support measure**	**Child physical activity measure**	**Results**	**Corrected effect size**
Beets et al. (2007) [142] USA	N = 259	CS	Child report	Child self-report	Girls’ MVPA & paternal support: β = −.09	-.11
	All female		Parental support – aggregated measure (encouragement, co-activity, transportation, watch, praise)	.70*	Girls’ MVPA & maternal support: β = .25	.32
	15.5 yrs		Mother α = .85			
			Father α = .91			
Brunet et al. (2014) [143] Canada	N = 558	CS	Child report	Accelerometer (7 d)	Girls’ MVPA & paternal support: r = .24, p < .001 (normal weight)	.28
	306 m, 252 f		Parental support – aggregated measure (co-activity, watch, transportation, encouragement, inform)	.80*	Girls’ MVPA & paternal support: r = .20	.19
	9.6 yrs		α = .77		**Mean r = .22**	
					Girls’ MVPA & maternal support: r = .10	
			ICC = .81		Girls’ MVPA & maternal support: r = .20 (normal weight)	
					**Mean r = .15**	
Butcher et al. (1983) [144] Canada	N = 696	CS	Self-report	Child self-report	Girls’ PA & maternal support: r = .21	.30
	All female		Parental support	.70*		
	11.0-16.0 yrs		.70*			
Butcher (1985) [145] Canada	N = 140	PRO	Child report	Child self-report	Girls’ PA (structured PA outside of school) & paternal support: r = .36, p < .01	.51
	All female	(5 yrs)	Parental support	.70*	Girls’ PA (structured PA outside of school) & maternal support: r = .27, p < .01	.39
	Followed from age 11.0 to 15.0 yrs		.70*			
Davison et al. (2003) [94] USA	N = 180	CS	Parent report	Child self-report	Girls’ PA & paternal	.19
	All females		Logistic support	PA measure a composite score of CPA-short, an activity checklist, & PACER	logistic support: r = .14	.28
	9.0 yrs		Mother α = .61	.70*	Girls’ PA & maternal logistic support: r = .18, p < .05	
			Father α = .74			
Hinkley et al. (2012) [100] Australia	N = 705	CS	Parent report	Accelerometer (8 d)	Girls’ PA & paternal logistic support: OR = 1.01, 95% CI .99-1.03	.01
	366 m, 262 f		Logistic support .70*	.80*		
	4.5 yrs					
Kirby et al. (2011) [146] UK	N = 641	PRO (5 yrs)	Child report	Child report	Girls’ PA & paternal support (yr 1): OR = 2.02, 95% CI 1.13-3.60 (p < .05)	.34
	313 m, 328 f		Parental support – aggregated measure (encouragement, transportation, watch, co-activity, praise)	PAQ-C	Girls’ PA & paternal support (yr 3): OR = 2.21, 95% CI 1.32-3.70 (p < .05)	.37
	Followed from age		.70*	.70*	Girls’ PA & paternal support (yr 5): OR = 1.98, 95% CI 1.06-3.71 (p < .05)	
	11-15 yrs				**Mean r = .24**	
					Girls’ PA & maternal support (yr 1): OR = 2.52, 95% CI 1.41-4.50 (p < .05)	
					Girls’ PA & maternal support (yr 3): OR = 2.21, 95% CI 1.32-3.70 (p < .05)	
					Girls’ PA & maternal support (yr 5): OR = 1.39, 95% CI = 0.54-3.58	
					**Mean r = .26**	
Raudsepp (2006) [110] Estonia	N = 329	CS	Parent report	7DPAR	Girls’ MVPA & fathers’ logistic support: r = .32, p < .010	.43
	168 m, 161 f		Parental logistic support –aggregated measure (enrollment, financial support, transportation)	.70*	Girls’ MVPA & mothers’ logistic support: r = .22, p < .01	.32
	13.8 yrs		Father α = .78			
			Mother α = .68			
Stucky-Ropp & DiLorenzo (1993) [147] USA	N = 242	CS	Parent report	Child self-reported	Girls’ PA & maternal support: r = .32	.46
	121 m, 121 f		Parental support – aggregated measure (encouragement, offers to exercise with child)	.70*		
	11.2 yrs		.70* (all respondents mothers)			

*Note.* *reliability not reported; 7DPAR = 7-day physical activity recall; CS = cross-sectional; d = days; f = females; m = males; MVPA = moderate to vigorous physical activity; PA = physical activity; PAQ-C = Physical Activity Questionnaire for Older Children; PRO = prospective.

### Parental modeling as a correlate

#### Overall effect size

A total of 36 effect sizes were used in the analysis to determine the overall relationship between parent and child PA (Table 8). Based on the fixed effects model and correcting for measurement error, parent and child PA associations approached a medium effect size (*r* = .29, 95% CI .28-.30). However, the results showed that the effect sizes in the sample were significantly heterogeneous *Q* (36) = 1597.52, *p* < .001. Due to the high degree of heterogeneity, using the point estimate from random effects model was appropriate, which resulted in a small effect size (*r* = .16, 95% CI .09-.24). Moreover, 98% of the observed variance was explained by true systematic effect size differences between studies.

**Table 8:** **Summary statistics for hypothesized moderators of children’s physical activity and parental modeling; fixed and random effects analyses (using corrected r values)**

**Variable**	**Q**_**b**_	**p**	**k**	**Random effects**		**Fixed effects**		**SE**	**Q**_**w**_	**I** ^**2**^
				**r**	**95% CI**	**r**	**95% CI**			
Parental modeling-summary			36	.16	.09-.24	.29	.28-.30	.04	1597.52*	97.81
**Developmental age**										
2-5.4 yrs	2.61	.27	9	.25	.06-.42	.20	.15-.24	.04	109.03*	90.70
5.5-12.4 yrs			17	.17	.09-.40	.08	.06-.10	.03	378.48*	94.65
12.5-19 yrs			10	.08	-.07-.22	.32	.32-.33	.05	467.01*	85.92
**Measurement of PA**										
Objective	1.42	.23	11	.24	.09-.37	.12	.08-.16	.04	115.80*	91.36
Reported			25	.13	.04-.22	.29	.29-.30	.04	1404.20*	98.29
**Quality**										
High	4.73	.09	4	-.05	-.26-.15	-.07	-.14-.00	.04	26.49*	88.67
Moderate			27	.19	.10-.27	.29	.29-.30	.04	1419.74*	98.17
Low			5	.22	.02-.40	.25	.19-.31	.04	31.58*	87.33
**Geographical location** ^**a**^										
USA	3.52	.06	24	.19	.09-.28	.32	.31-.32	.06	889.55*	97.41
AUS/NZ			6	.02	-.13-.17	.00	-.03-.03	.03	36.11*	86.15
Study design										
CS	.56	.45	32	.15	.07-.23	.30	.30-.31	.04	1210.09*	97.44
PRO			4	.23	.04-.41	.02	-.01-.05	.04	28.79	89.58

*Note:* *p < .001; ^a^some countries excluded from the analysis based on < 4 effect sizes.

#### Moderators of child physical activity

Table 8 indicates that subsequent analyses did not find any of the proposed moderators of parent and child physical activity to be significant (*p* > .05).

Based on 49 effect sizes, our analyses found that parental gender moderated the relationship between boys’ PA and parents’ PA (Table 9). The results showed that father-son PA (*r* = .29, 95% CI .21-.36) was significantly higher than mother-son PA (*r* = .19, 95% CI .14-.23; p < .05). For parental modeling and girls’ PA, results from the 62 effect sizes showed that parental gender did not moderate the relationship. The correlation for father-daughter PA (r = .22, 95% CI 16-.27) and mother-daughter PA (r = .23, 95% CI .18-.27) were both similar in magnitude.

**Table 9:** **Summary statistics for parent–child intergenerational relationships for boys’ and girls’ physical activity and parental modeling; fixed and random effects analyses (using corrected r values)**

**Variable**	**Q**_**b**_	**p**	**k**	**Random effects**		**Fixed effects**		**SE**	**Q**_**w**_	**I** ^**2**^
				**r**	**95% CI**	**r**	**95% CI**			
**Parental modeling**										
Son-father	4.89	.03	24	.29	.21-.36	.27	.25-.29	.02	386.57*	94.05
Son-mother			25	.19	.14-.23	.15	.13-.16	.01	160.91*	85.09
Daughter-father	.05	.83	28	.22	.16-.27	.21	.19-.22	.01	288.48*	90.64
Daughter-mother			34	.23	.18-.27	.22	.21-.24	.01	352.81*	90.65
**Parental support**										
Daughter-father	.26	.61	7	.24	.07-.40	.22	.17-.26	.03	71.68*	91.63
Daughter-mother			8	.32	.27-.37	.32	.28-.35	.00	10.64	34.23

*Note:* *p < .001.

### Parental support as a correlate

#### Overall effect size

A total of 34 effect sizes were used to estimate the relationship between overall parental support and child PA (Table 10). Both the fixed and random effects model found that the relationship between parental support and child PA was moderate in size (*r* = .38). Analyses from the fixed model also indicated that a significant degree of heterogeneity within the sample was present (*Q* (34) = 1204.70, *p* < .001) and that 97% of the observed variance was explained by true systematic effect size differences between studies.

**Table 10:** **Summary statistics for hypothesized moderators of children’s physical activity and parental support; fixed and random effects analyses (using corrected r values)**

**Variable**	**Q**_**b**_	**p**	**k**	**Random effects**		**Fixed effects**		**SE**	**Q**_**w**_	**I** ^**2**^
				**r**	**95% CI**	**r**	**95% CI**			
**Parental support**										
Composite-summary			34	.38	.30-.46	.38	.37-.39	.03	1204.70*	97.26
Coactivity			12	.28	.03-.50	.29	.27-.31	.13	2237.45*	99.51
Encouragement			19	.34	.25-.41	.36	.34-.37	.02	537.72*	96.65
Praise			6	.15	-.03-.32	.15	.11-.19	.04	88.49*	94.35
Watching			5	.16	.05-.27	.13	.10-.16	.01	30.47*	86.87
Transportation			6	.22	.12-.31	.16	.14-.19	.01	38.08*	86.87
Equipment			12	.21	.15-.27	.21	.17-.24	.01	32.49*	66.14
Monitoring			4	.14	-.03-.30	.22	.17-.27	.03	18.33*	83.64
**Developmental age**										
Overall/composite										
2-5.4 yrs	2.02	.37	7	.30	.18-.41	.28	.24-.33	.02	36.83*	83.71
5.5-12.4 yrs			10	.35	.21-.47	.38	.37-.40	.04	328.95*	97.26
12.5-19 yrs			17	.42	.29-.55	.40	.38-.41	.05	818.32*	98.05
Encouragement										
2-5.4 yrs	0.48	.79	5	.29	.10-.45	.14	.10-.18	.04	30.90*	87.05
5.5-12.4 yrs			8	.36	.23-.48	.31	.28-.34	.03	103.89*	93.26
12.5-19 yrs			6	.34	.21-.45	.41	.40-.43	.03	213.24*	97.66
**Measurement of PA**										
Overall/composite										
Objective	19.53	<.01	11	.20	.13-.26	.21	.18-.24	.01	29.97	66.63
Self-report			23	.46	.37-.55	.41	.40-.43	.03	1047.24*	97.90
Encouragement										
Objective	.00	.98	7	.34	.14-.51	.34	.27-.40	.05	38.83*	84.55
Reported			12	.34	.24-.43	.36	.34-.37	.02	498.52*	97.79
Quality										
Overall/composite							.			
High	.57	.45	7	.48	.17-.71	.38	36-.41	.18	659.51*	99.09
Moderate/low			24	.37	.29-.44	.41	.40-.43	.02	445.01*	94.83
Geographical Location^a^										
Overall/composite										
USA	2.80	.25	21	.33	.25-.41	.37	.36-.39	.04	408.09*	95.10
AUS/NZ			4	.59	-.14-.90	.69	.66-.73	.68	438.27*	99.32
EUR			6	.45	.32-.56	.40	.37-.43	.03	75.55*	93.38
Encouragement										
USA	2.87	.09	13	.36	.27-.44	.33	.30-.35	.03	105.40*	88.62
EUR			4	.20	.04-.35	.28	.26-.30	.03	125.39*	97.61

*Note:* *p < .001; ^a^some countries excluded from the analysis based on < 4 effect sizes.

According to the corrected random effects models, many of the effect sizes for the various individual support behaviours were small. Parent–child co-activity, praising the child for being active, watching the child participate in PA, providing transportation to a place where the child could be active, monitoring the child’s PA levels, and supplying the child with PA equipment ranged between *r* = .15-.28 (Table 10). The only support behaviour to have a moderate effect size was the relationship between parental encouragement and child PA (*r* = .34, 95% CI .25-.41). Overall, the dispersal of the effect sizes calculated was variable, ranging from 66 to 100%.

#### Moderators of child physical activity

Table 10 presented the potential moderators that were investigated in our analysis. In the analysis, child and adolescent PA was moderated by the type of measurement used to quantify the child’s PA (*p* < .001). When objective PA measures were used, the results showed a small effect of *r* = .20 (95% CI .13-.26) between a composite measure of parental support and child PA; whereas reported PA had a moderate effect size of *r* = .46 (95% CI .37-.55). Developmental age, study design, and geographical location were not significant moderators of overall parental support and child PA. Due to the limited number of prospective studies, moderator analyses were not conducted to examine the effects of study design.

Among individual supportive behaviours, only parental encouragement had an adequate amount of studies to examine potential moderating variables (Table 10). Moderating variables such as developmental age and geographical location were not significant moderators of the parental encouragement and child PA relationship (*p* > .05).

When examining the relationship between girls’ PA and parental support, the summary analysis of 10 effect sizes found that the parental gender did not significantly moderate this relationship (p > .05) (Table 9). Analyses exploring the moderating effects of parental gender in boys’ PA were limited by the number of studies and were not conducted.

### Publication bias

Funnel plots were constructed to investigate the possibility of publication bias for parent and child PA, parental support and child PA, and individual support behaviours and child PA associations. When visually inspected, the resulting funnel plots suggested a potential publication bias for parent and child PA, and providing transportation for the child to be active and child PA associations.

A subsequent classic fail-safe N analysis for child–parent PA associations showed that 7590 studies with a mean effect of zero were necessary for the overall effect found to become statistically insignificant. Based on this relatively large computation, it indicated that the results were not skewed. However, for providing the child with transportation to opportunities to be active, only 198 studies needed to create a mean effect of zero for the effect to be insignificant, alluding to a skewed effect size. Subsequent trim and fill analyses specified that it was necessary to trim two studies from the computation. With the correction, the effect size for transporting the child to physical activities and child PA decreased from the original point estimate of *r* = .22 (95% CI .12-.31) to a corrected point estimate of *r* = .14 (95% CI .03-.24).

## Discussion

The main objectives of this meta-analysis were to thoroughly investigate and quantify the strength of parental correlates and identify whether parent–child gender interactions are notable in child and adolescent PA. Previous systematic reviews have been narrative in nature and meta-analyses attempting to quantify the overall effects between parental support and modeling behaviours and child PA have been restricted to 20–30 studies [8,16] – resulting in a partial depiction of the parental correlates in child and adolescent PA. This meta-analysis encompasses 112 studies published to date and thus sheds a more definitive light on the relationship between parental behaviours and children’s PA.

One of the contentious topics has been whether parental modeling is an important correlate in child and adolescent PA. Recent narrative reviews have suggested that parent’s PA behaviours were unassociated with child and adolescent PA [14,20]. The meta-analysis conducted by Pugliese and Tinsley [8] found a small effect (*r* = .10) for parent and child PA. Our results, after correcting for measurement error, concurred with the previous meta-analysis showing a small overall association between parental and child PA.

During preadolescent years, parental modeling of PA plays an integral role in establishing a social norm regarding activity [7], but as the child matures, modeling behaviours in the PA domain may be drawn from the emergent influence of the child’s peers while the influence of parental modeling wanes. It is also possible that in early years of childhood parent–child coactivity is more prevalent; and as the child ages, the association between parent and child PA bifurcates and becomes more independent from each other. In any case, the results suggest the importance of family-based coactivity interventions in the early years of child development.

A number of narrative reviews have consistently identified an association between parental support and children’s PA [6-9,11,12,14,16,18-21]. This meta-analysis is the first to quantify the relationship between overall parental support and child PA as well as various individual supportive behaviours. In our analyses, overall parental support and child PA yielded a medium effect size. This effect is worthy of noting, particularly when compared to other correlates of child behaviour. For example, a recent meta-analysis examining children’s affective judgments in PA, found that affect had a small to medium an effect size (*r* = .26) between children’s affect and PA behaviour [25]. Based on these findings, it suggests that parental support for child and adolescent PA may be an important consideration for future PA intervention efforts.

In line with this thinking, it is important to examine whether any particular support behaviour is of critical value over others as a potential intervention target. Our analyses of specific behaviours such as praising the child, watching the child participate in PA, engaging in parent–child co-activity, transporting the child to places where the child could be active, and providing the child with equipment all had small effect sizes (r = .14-.28). The only individual support behaviour that was moderate in size was parental encouragement. To date, much quantitative reviews have only investigated the individual support behaviour of parental encouragement on child PA, which has been identified as a small correlation of *r* = .15-.18 [8,16], which is smaller than our results. However, it is important to mention that these correlations were not previously corrected for measurement error. Overall, based on these various small effect sizes, it may be important to consider the potency of parental support taken as an aggregate rather than any individual support behaviour.

To date, various studies have examined the moderating effect of parental gender in boys’ and girls’ PA, yet the finding has been unclear and speculative. In a systematic review, a positive association was found for father-son PA [7]. Similarly, among maternal relationships, mother-daughter PA was significantly related [7]. Our results brought forth a degree of transparency regarding parent–child gender interactions further supporting a stronger correlation for father-son PA. However, in our results no differences were found for mother-son and mother-daughter PA correlations. In the area of parental support, no differences were found for the maternal and paternal interaction for girls’ PA. However, the parental interaction regarding boys’ PA will require further investigation. Overall, these results suggested that the importance between the intergenerational relationship between father and son PA and may be an important consideration when targeting boys’ activity behaviour. As well, our findings indicated that the incorporation of parental support behaviours, irrespective of parental gender, were an essential component for prospective interventions that target girls’ PA.

The limitations of this review highlight the fact that additional research is needed in several areas to improve our understanding of the correlates in child and adolescent PA. First, the use of parental support instruments and reporting of the correlation between parental support and child PA in this meta-analysis have been quite diverse, which also has been documented in the previously published literature [18,22]. Moving forward, it may be important to utilize previously validated measures, such as the activity support scale [37], and report both individual support behaviours and parental support as a construct (see [22] for an overview of parental support measures). Second, an important consideration may be children’s peers and siblings and how they relate the child’s behaviour. Thus, future research will be needed to explore the role of socialization of the immediate social network outside of the family unit and whether other children provide more salient models or social support for PA. Third, much of the research has been limited to developed nations such as the United States, Australia, or Europe. More studies will be needed from other countries to explore whether cultural differences are present. Fourth, an important detail to underscore from this review was that many of the parental respondents were mothers. It may be important to investigate the roles of fathers in the area of parental support and child PA. Lastly, several individual support behaviours were unexamined due to the limited amount of research (e.g., informing the child that PA is beneficial or financial support). More research is needed to uncover the relationship these support behaviours and child PA and whether certain parental support behaviours are conducive to a specific type of PA (e.g., structured or unstructured PA).

In summary, this meta-analysis presents results that align with previous reviews but represent a larger and more robust assessment of the parental and child correlates literature and the consideration for measurement error and methodologic quality. The findings demonstrate that both parental modeling and support related to child and adolescent PA. However, overall parental support emerged as a sizeable correlate linked to child activity. In addition to this, our results revealed a significant degree of heterogeneity among the studies that could not be explained well by our proposed moderators. In order to advance our intervention approaches to increase PA in children and adolescents, it will be critical to consider the development of interventions based on the child’s developmental age. More notably, it will be essential to integrate parents as a source of social support to change child and adolescent PA behaviour.

## Abbreviation

PA: Physical activity
